# Prediction of non-perfusion volume ratio for uterine fibroids treated with ultrasound-guided high-intensity focused ultrasound based on MRI radiomics combined with clinical parameters

**DOI:** 10.1186/s12938-023-01182-z

**Published:** 2023-12-13

**Authors:** Ye Zhou, Jinwei Zhang, Chenghai Li, Jinyun Chen, Fajin Lv, Yongbin Deng, Siyao Chen, Yuling Du, Faqi Li

**Affiliations:** 1 State Key Laboratory of Ultrasound in Medicine and Engineering, College of Biomedical Engineering, Chongqing Medical University, Chongqing, 400016 China; 2 Chongqing Key Laboratory of Biomedical Engineering, Chongqing Medical University, Chongqing, 400016 China; 3 Department of Radiology, The First Affiliated Hospital of Chongqing Medical University, Chongqing, 400016 China; 4 Chongqing Haifu Hospital, Chongqing, 401121 China

**Keywords:** Radiomics, Magnetic resonance imaging, Uterine fibroid, Ultrasound guided high-intensity focused ultrasound, Prediction, Machine learning

## Abstract

**Background:**

Prediction of non-perfusion volume ratio (NPVR) is critical in selecting patients with uterine fibroids who will potentially benefit from ultrasound-guided high-intensity focused ultrasound (HIFU) treatment, as it reduces the risk of treatment failure. The purpose of this study is to construct an optimal model for predicting NPVR based on T2-weighted magnetic resonance imaging (T2MRI) radiomics features combined with clinical parameters by machine learning.

**Materials and methods:**

This retrospective study was conducted among 223 patients diagnosed with uterine fibroids from two centers. The patients from one center were allocated to a training cohort (*n* = 122) and an internal test cohort (*n* = 46), and the data from the other center (*n* = 55) was used as an external test cohort. The least absolute shrinkage and selection operator (LASSO) algorithm was employed for feature selection in the training cohort. The support vector machine (SVM) was adopted to construct a radiomics model, a clinical model, and a radiomics–clinical model for NPVR prediction, respectively. The area under the curve (AUC) and the decision curve analysis (DCA) were performed to evaluate the predictive validity and the clinical usefulness of the model, respectively.

**Results:**

A total of 851 radiomic features were extracted from T2MRI, of which seven radiomics features were screened for NPVR prediction-related radiomics features. The radiomics–clinical model combining radiomics features and clinical parameters showed the best predictive performance in both the internal (AUC = 0.824, 95% CI 0.693–0.954) and external (AUC = 0.773, 95% CI 0.647–0.902) test cohorts, and the DCA also suggested the radiomics–clinical model had the highest net benefit.

**Conclusions:**

The radiomics–clinical model could be applied to the NPVR prediction of patients with uterine fibroids treated by HIFU to provide an objective and effective method for selecting potential patients who would benefit from the treatment mostly.

**Supplementary Information:**

The online version contains supplementary material available at 10.1186/s12938-023-01182-z.

## Introduction

Ultrasound-guided high-intensity focused ultrasound (HIFU) is a noninvasive ablation option for patients with uterine fibroids with the advantages of fast recovery, low complication occurrence, and uterus preservation, and therefore, it has been increasingly applied worldwide [1, 2]. As an ablation technique, the efficacy of HIFU is closely related to the immediate postoperative non-perfused volume ratio (NPVR). A higher NPVR heralds a significant improvement in the patient’s symptoms, and an NPVR of over 80% is considered as an indicator of therapeutic success in recent studies [3, 4]. However, not all patients can benefit from this treatment because of several limitations, such as high cellularity and high vascularity in the fibroids, which will hinder the deposition of ultrasonic energy and make ablation outcomes unsatisfactory. How to determine suitable patients for HIFU treatment has become one of the major challenges in its clinical application [5, 6]. In clinical practice, the Funaki classification base on T2-weighted magnetic resonance imaging (T2MRI) is widely used to determine whether a patient is suitable for HIFU treatment. The hyper-intensity (type III in Funaki classification: the signal intensity of fibroids is equal to or greater than that of the myometrium) usually indicates poor treatment response because of greater cellularity and vascularity of fibroids, making propagation of acoustic energy difficult. While those hypointense (Funaki I) and isointense (Funaki II) fibroids with signal intensity is lower than the muscle layer or skeletal muscle on T2MRI have a significantly higher nonperfused volume (NPV) after ablation [7, 8]. Current the qualitative assessment based on T2MRI, however, can only provide limited guidance for personalized clinical treatment decisions, since adequate quantification of fibroid heterogeneity is lacking. In addition, some other factors, such as the location and diameter of the fibroids, will also affect the results of the ablation. Therefore, traditional classification based solely on T2MRI signal intensity may not be able to accurately predict treatment outcome [4, 9].

Some studies are devoted to obtaining more accurate NPVR predictors from clinical parameters and quantitative perfusion parameters. Suomi [10] established a prediction model through support vector machine (SVM) based on clinical parameters—including subcutaneous fat thickness, fibroids size, and distance from the fibroids to the skin to predict the NPVR value (> 80%), and the maximum F1-micro classification score was 0.63, which indicates that that model based on clinical parameters alone has poor prediction performance, the possible reason is that the influence of blood perfusion is ignored. The quantitative perfusion parameter *K*^trans^ can illustrate the vascular permeability and is negatively correlated with NPVR, was consequently considered as an important prediction indicator in poor therapeutic response [11]. Wei [12] and Li [13] predicted the NPVR (> 70% and > 60%) using *K*^trans^ value, with AUC values of 0.803 and 0.817, respectively. Keserci [14] further combined clinical parameters with semi-quantitative perfusion parameters to predict NPVR (> 90%), and its AUC was 0.948. Blood perfusion parameters have shown potential in accurately predicting the NPV ratio; however, dynamic-enhanced magnetic resonance imaging (DCE–MRI) has high requirements for image acquisition and post-processing expertise and is, therefore, not routinely used in clinical practice [13, 15]. A personalized prediction method that is quantitative, objective and easy to clinically implement is an urgent need for HIFU clinical practice.

Radiomics has been applied to various therapeutic response predictions for tumors, such as non-small cell lung cancer, pancreatic cancer, and rectal cancer [16–18]. Such applications arise from the ability of radiomics to transform medical images into high-dimensional quantitative information, so as to quantify the heterogeneity of tumors, capturing subtle structural differences that cannot be detected by human vision [19]. A recent study employed a radiomics model based on T2-weighted imaging (T2WI) to predict the prognosis of uterine fibroids treated with HIFU [20]. The results suggest that radiomics is a potential approach for predicting the efficacy of HIFU. Compared with subjective evaluation, radiomic analysis is more stable and objective. However, there is still a lack of reports on the use of radiomics to predict the NPVR in the HIFU treatment of uterine fibroids.

Here we established a prediction model based on T2MRI radiomics features along with clinical parameters through machine learning, aiming to provide an approach for predicting the NPVR of uterine fibroids treated by HIFU.

## Results

### Patients

**Fig. 1:**
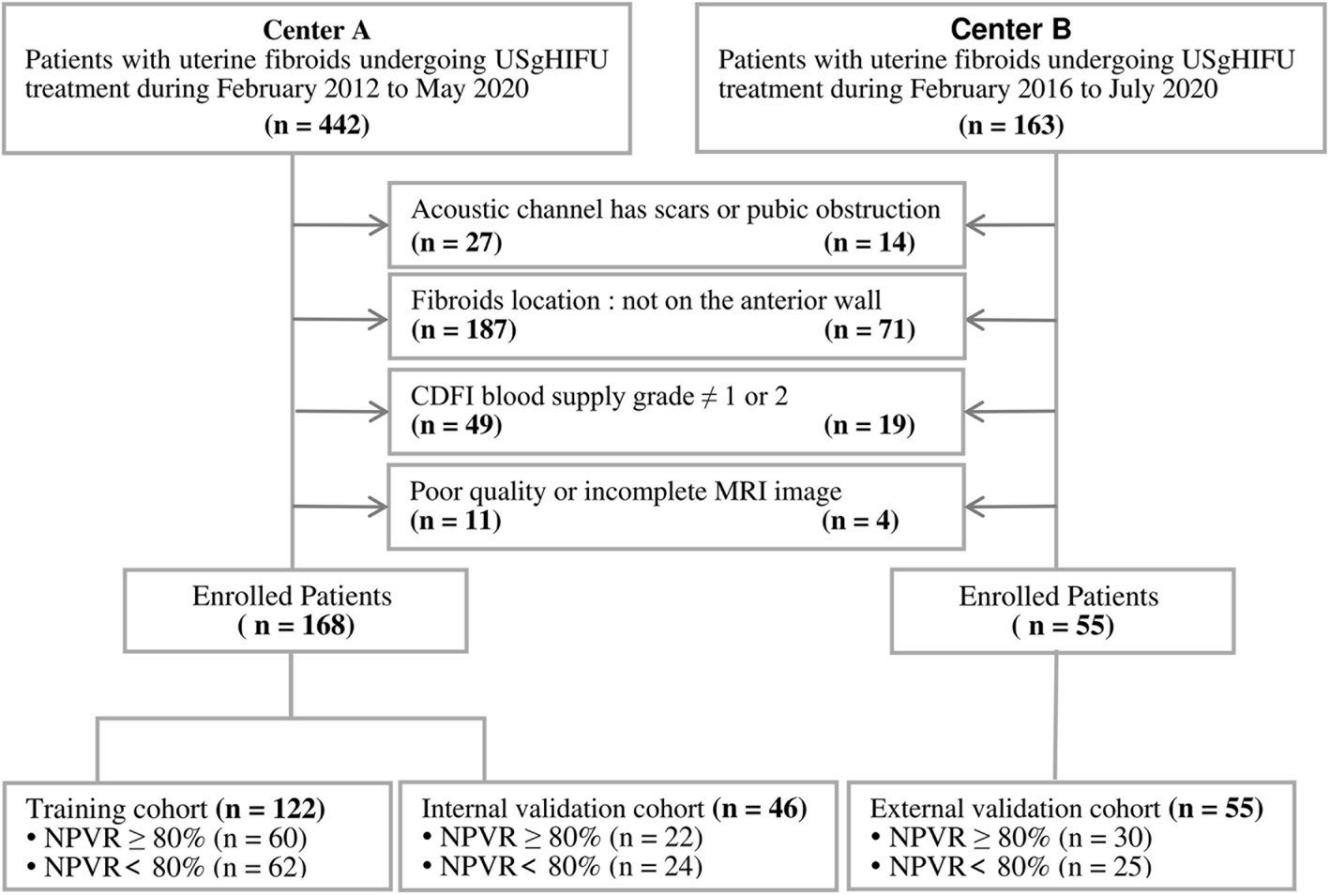
Process of patient inclusion and exclusion. **Center A**: First Affiliated Hospital of Chongqing Medical University. **Center B**: Chongqing Haifu Hospital

The patient screening process is shown in Fig. 1. Among all 605 patients, 223 patients were selected for this study, the most frequent reason (67.5%) for the exclusion of participants was that the fibroids were not located on the anterior wall. There was no instance involving the cancellation of HIFU treatment because of technical failure.

The baseline characteristics of the patients are listed in Table 1. The signal intensity on T2MRI was significantly different between H and L groups (*P* < 0.05) in the training and internal test cohorts, and no significant differences were found in other characteristics. The mean NPVR of patients in the training cohort was 75.6% (Group H: 89.2%, Group L: 61.2%), 76.9% in the internal test cohort (Group H: 90.3%, Group L: 63.2%), and 78.1% in the external test cohort (Group H: 89.5%, Group L: 62.6%).

**Table 1:** Baseline characteristics of the study population

Characteristics	Training (*n* = 122)		*P* value	Internal validation (*n* = 46)		*P* value	External validation (*n* = 55)		*P* value
	Group H (*n* = 60)	Group L (*n* = 62)		Group H (*n* = 22)	Group L (*n* = 24)		Group H (*n* = 30)	Group L (*n* = 25)	
Age, years	41.0 ± 6.4	39.1 ± 6.5	0.121	39.6 ± 5.1	37.7 ± 6.1	0.793	39.1 ± 6.2	38.1 ± 8.6	0.196
Subcutaneous fat thickness (mm)	15.5 ± 5.5	17.1 ± 5.3	0.112	15.6 ± 5.1	16.2 ± 5.2	0.882	16.0 ± 3.7	17.2 ± 4.7	0.398
Thickness of the rectus abdominis (mm)	8.8 ± 2.4	8.1 ± 2.9	0.619	8.5 ± 3.6	8.1 ± 2.8	0.681	10.2 ± 2.7	10.6 ± 2.7	0.268
Diameter of UFs (cm)	5.3 ± 1.4	5.8 ± 1.4	0.062	5.7 ± 1.9	6.2 ± 2.2	0.361	5.3 ± 1.2	5.9 ± 1.5	0.152
Volume of UFs (cm^3^)	93.1 ± 75.7	117.7 ± 90.7	0.155	104.3 ± 117.8	139.1 ± 118.9	0.162	110.3 ± 82.2	132.6 ± 92.1	0.302
Distance (mm)^a^	92.4 ± 16.8	97.9 ± 15.6	0.062	91.1 ± 17.4	99.7 ± 13.8	0.069	93.1 ± 13.1	100.5 ± 11.7	0.095
Number of fibroids (total)			0.909			0.887			0.702
1	45 (75.0%)	49 (79.1%)		16 (78.6%)	17 (72.4%)		21 (72.4%)	20 (76.9%)	
2–5	15 (25.0%)	13 (20.9%)		6 (21.4%)	7 (27.6%)		8 (27.6%)	6 21.1%)	
SI on T2MRI^b^			0.000*			0.039*			0.016*
Type 1	27 (45.0%)	17 (27.4%)		10 (45.5%)	6 (25.0%)		12 (40.0%)	7 (28.0%)	
Type 2	27 (45.0%)	20 (32.3%)		8 (36.4%)	4 (16.6%)		12 (40.0%)	3 (12.0%)	
Type 3	2 (3.3%)	19 (30.7%)		3 (13.6%)	7 (29.2%)		4 (13.3%)	11 (44.0%)	
Type 4	4 (6.7%)	6 (9.7%)		1 (4.5%)	7 (29.2%)		2 (6.7%)	4 (16.0%)	
CDFI grade^c^			0.297			0.821			0.761
Grade 1	21(35.0%)	16 (25.8%)		4 (18.2%)	5 (20.8%)		12 (40.0%)	9 (36.0%)	
Grade 2	39 (65.0%)	46 (74.2%)		18 (81.8%)	19 (79.2%)		18 (60.0%)	16 (64.0%)	
Fibroid types			0.209			0.659			0.561
Intramural	38 (63.3%)	48 (77.4%)		16 (72.7%)	20 (83.3%)		19 (63.4%)	13 (52.0%)	
Subserosal	8 (13.4%)	4 (6.5%)		2 (9.1%)	1 (4.2%)		4 (13.3%)	6 (24.0%)	
Submucosal	14 (23.3%)	10 (16.1%)		4 (18.2%)	3 (12.5%)		7 (23.3%)	6 (24.0%)	

Group H: NPVR ≥ 80%; Group L: NPVR < 80%

*UFs* uterine fibroids

*Significance indicated by *P* < 0.05

^a^Distance: Distance from the back end of the fibroids to the skin surface

^b^SI: signal intensity; Type 1: hypointense; Type 2: isointense; Type 3: homogeneous-type hyperintense; Type 4: heterogeneous-type hyperintense

^c^CDFI: Color Doppler Flow Imaging; Grade 1: a small amount of blood flow in the fibroids, 1–2 punctate blood flows can be seen; Grade 2: moderate blood flow in fibroids, 1 main blood vessel can be seen, whose length exceeds the radius of the fibroids or 2–3 small blood vessels can be seen

### Radiomics feature selection and Rad-score calculation

**Fig. 2:**
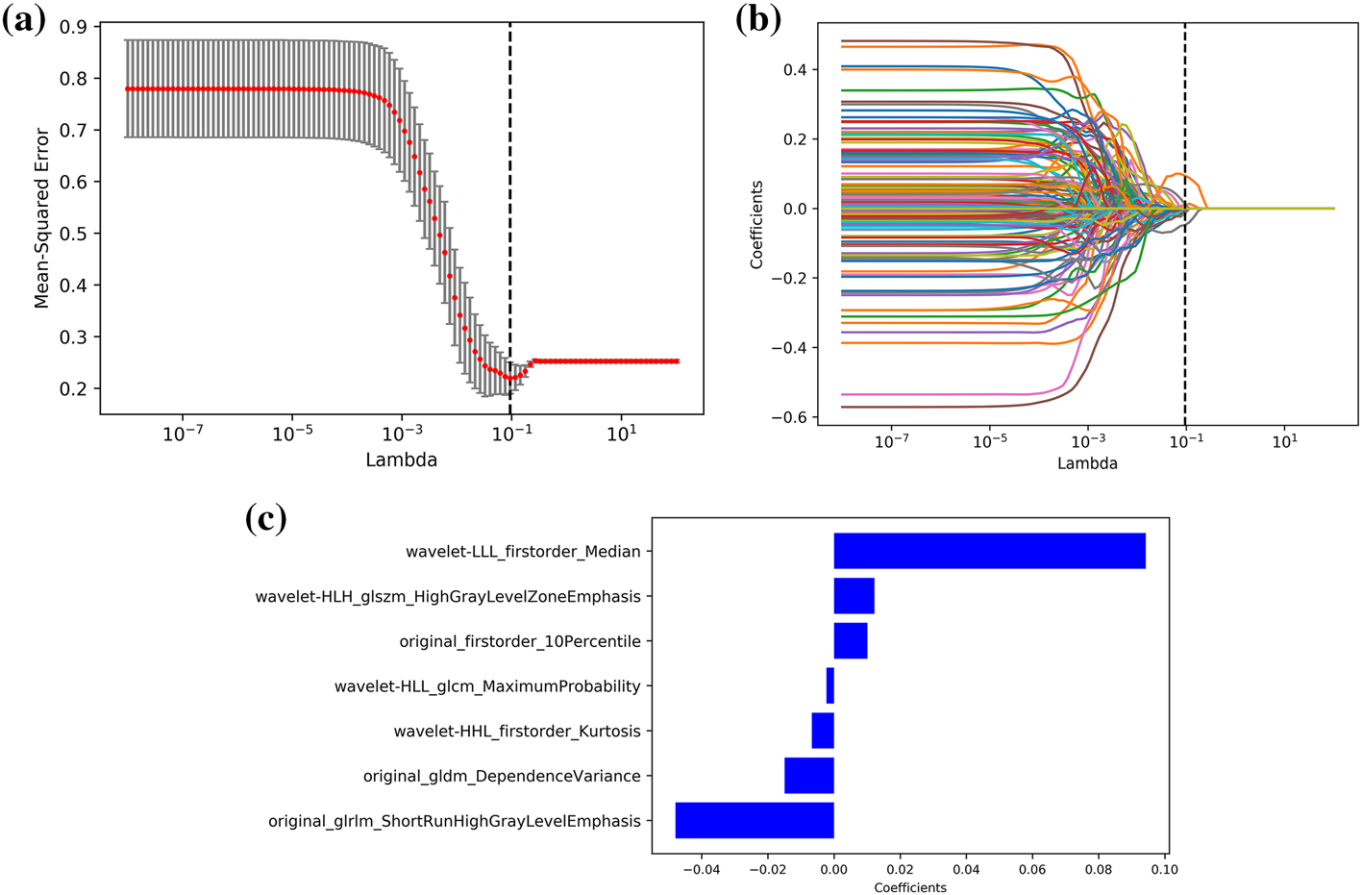
Radiomics feature selection. **a** Selection of the tuning parameter *λ* in the LASSO model via mean square error on each fold in tenfold cross-test method, dotted vertical lines show the optimal values using the minimum criteria. **b** LASSO coefficient profiles of the 851 radiomics features, resulting in 8 nonzero coefficient features. **c** The most predictive feature subsets selected by LASSO and their correlation coefficients

Of the 851 extracted radiomic features, 691 (81.2%) showed good interobserver agreement, with ICCs > 0.8. Among which 7 features with nonzero coefficients were selected using LASSO, namely, original_firstorder_10Percentile, original_glrlm_ShortRunHighGrayLevelEmphasis, original_gldm_DependenceVariance, wavelet-HLL_glcm_MaximumProbability, wavelet-HLH_glszm_HighGrayLevelZoneEmphasis, wavelet-HHL_firstorder_Kurtosis and wavelet-LLL_firstorder_Median. Figure 2 presents the LASSO feature selection process and the selected features as well as their coefficients.

Rad-score of each patient was calculated according to the selected features and their corresponding coefficients.

**Fig. 3:**
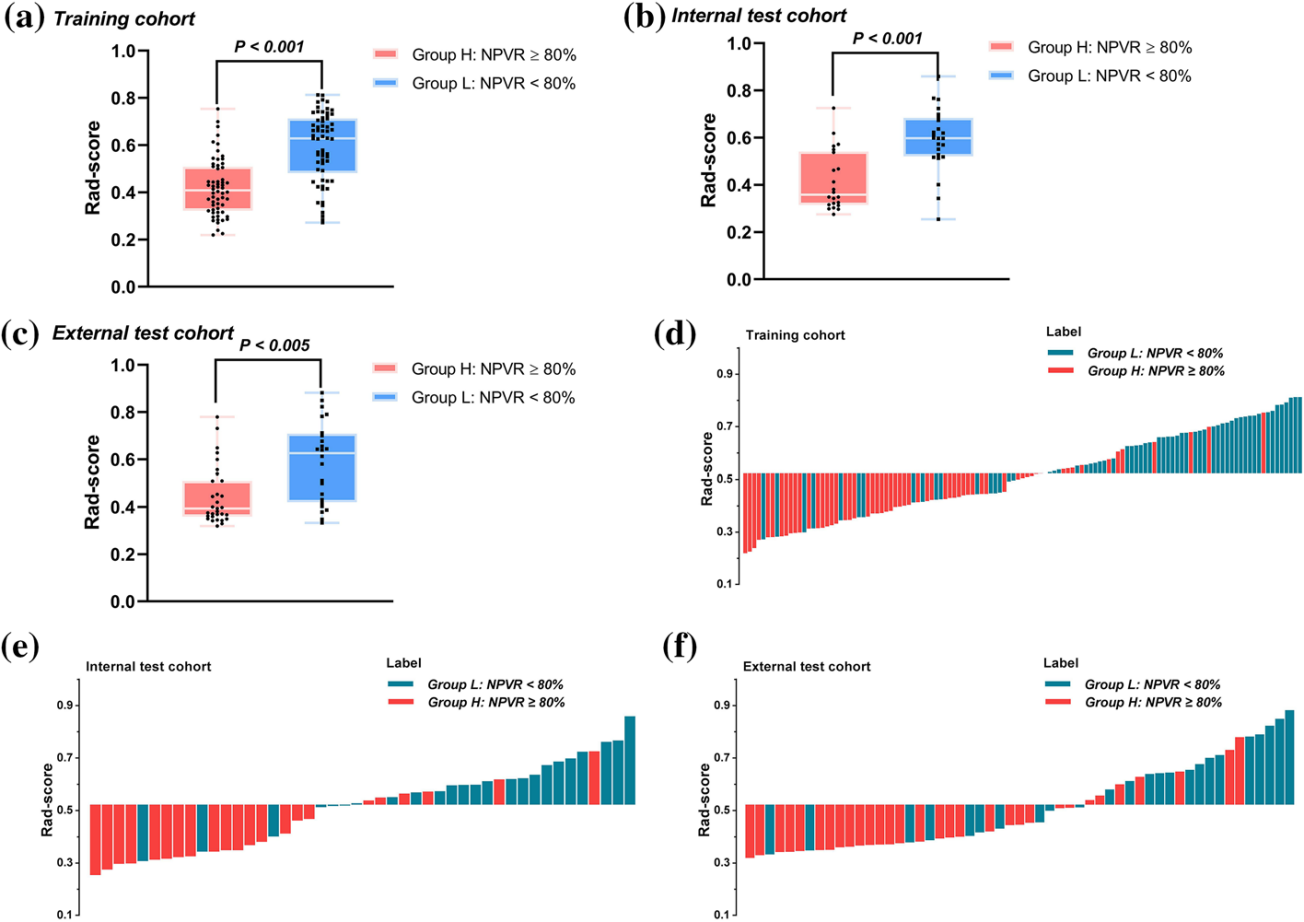
Radiomics score (Rad-score) boxplots of the 3 cohorts, each black dot representing the Rad-score of an individual in **a** the training cohort, **b** the internal cohort, and **c** the external cohort; **d**–**f** the distribution of Rad-scores in each cohort, with red bars representing patients with NPVR ≥ 80%, blue bars NPVR < 80%. The cutoff value of the radiomics score was 0.522

Figure 3a–c shows that the Rad-score of patients with NPVR ≥ 80% was significantly lower than that of patients with NPVR < 80% (training cohort), which was subsequently confirmed in the 2 test cohorts. In the training cohort, the optimal radiomics cutoff value of 0.522 was determined based on the maximum Youden index in the NPVR ≥ 80% group. Figure 3d–f represents the Rad-score distribution consisting of the Rad-score value of each patient in both training and the 2 test cohorts, which clearly revealed that almost all the patients’ NPVR could be predicted by the cutoff value of their radiomics features. The formula and statistical analysis of Rad-scores are present in Additional file 1: Equation S1 and Table S4.

### Model evaluation

**Fig. 4:**
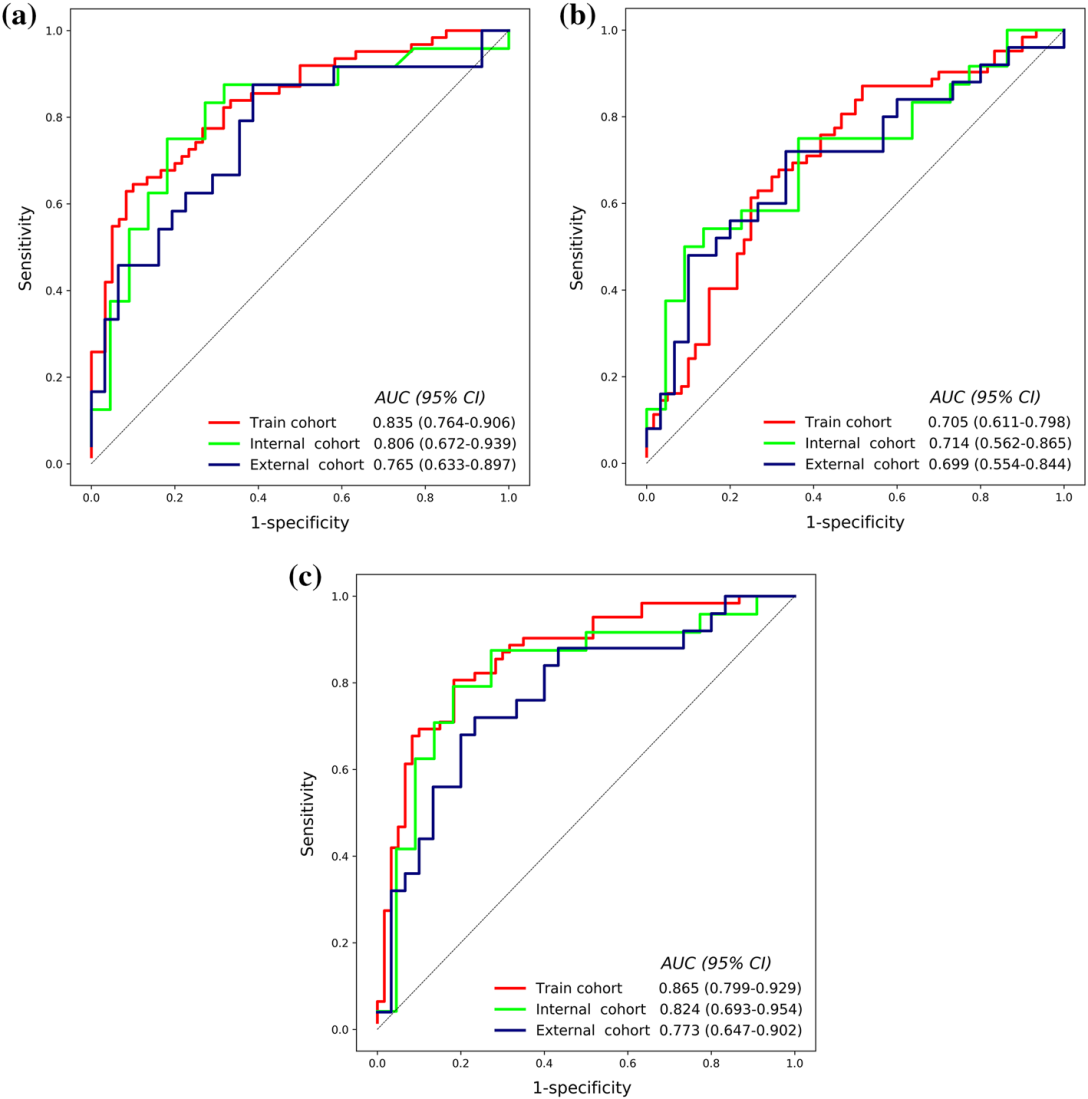
ROC curves of **a** radiomics model, **b** clinical model, and **c** radiomics–clinical model. The red, green, and blue curves representing the training cohort, internal test cohort, and external test cohort, respectively

The linear kernel function was chosen as the most suitable kernel function for all analyses in this study after tenfold cross-validation and grid search processing. The prediction performance of radiomics model, clinical model, and radiomics–clinical model are shown in Table 2. The radiomics–clinical combination model demonstrated the greatest predictive validity (Fig. 4).

**Table 2:** Predictive performance of radiomics model, clinical model, and radiomics–clinical model

Model	Training (*N* = 122)			Internal test (*N* = 46)			External test (*N* = 55)		
	Radiomics	Clinical	Combination	Radiomics	Clinical	Combination	Radiomics	Clinical	Combination
AUC (95% CI)	0.835 (0.76, 0.90)	0.705 (0.61, 0.79)	0.865 (0.79, 0.92)	0.806 (0.67, 0.93)	0.714 (0.56, 0.86)	0.824 (0.69 0.95)	0.765 (0.63, 0.89)	0.699 (0.55, 0.84)	0.773 (0.64, 0.90)
Sensitivity	71.2%	66.1%	76.6%	76.2%	66.7%	80.0%	72.4%	73.1%	75.0%
Specificity	76.8%	68.3%	81.3%	76.0%	64.3%	76.9%	61.5%	62.1%	66.7%
Accuracy	73.8%	67.2%	78.7%	76.1%	65.2%	78.3%	67.3%	67.3%	70.9%

The radiomics model based on the 7 radiomics features of T2MRI showed high performance in predicting NPVR, with an AUC of 0.806 (95% CI 0.672–0.939) and 0.765 (95% CI 0.633–0.897) in the internal and external test cohorts, respectively. The clinical model established on the 5 clinical parameters of subcutaneous fat thickness, distance from the posterior surface of fibroids to the skin surface, fibroids diameter, fibroids volume, and T2 signal intensity had a limited predictive capability, showing an AUC of 0.714 (95% CI 0.545–0.852) and 0.699 (95% CI 0.565–0.851) in the internal and external test cohorts. The patient’s clinical parameters were added to the radiomics features data set, and the radiomics–clinical combined model was obtained through SVM training. The combined model improved the AUC to 0.824 (95% CI 0.693–0.954) and 0.773 (95% CI 0.647–0.902), respectively, in the 2 test cohorts.

### Clinical application

**Fig. 5:**
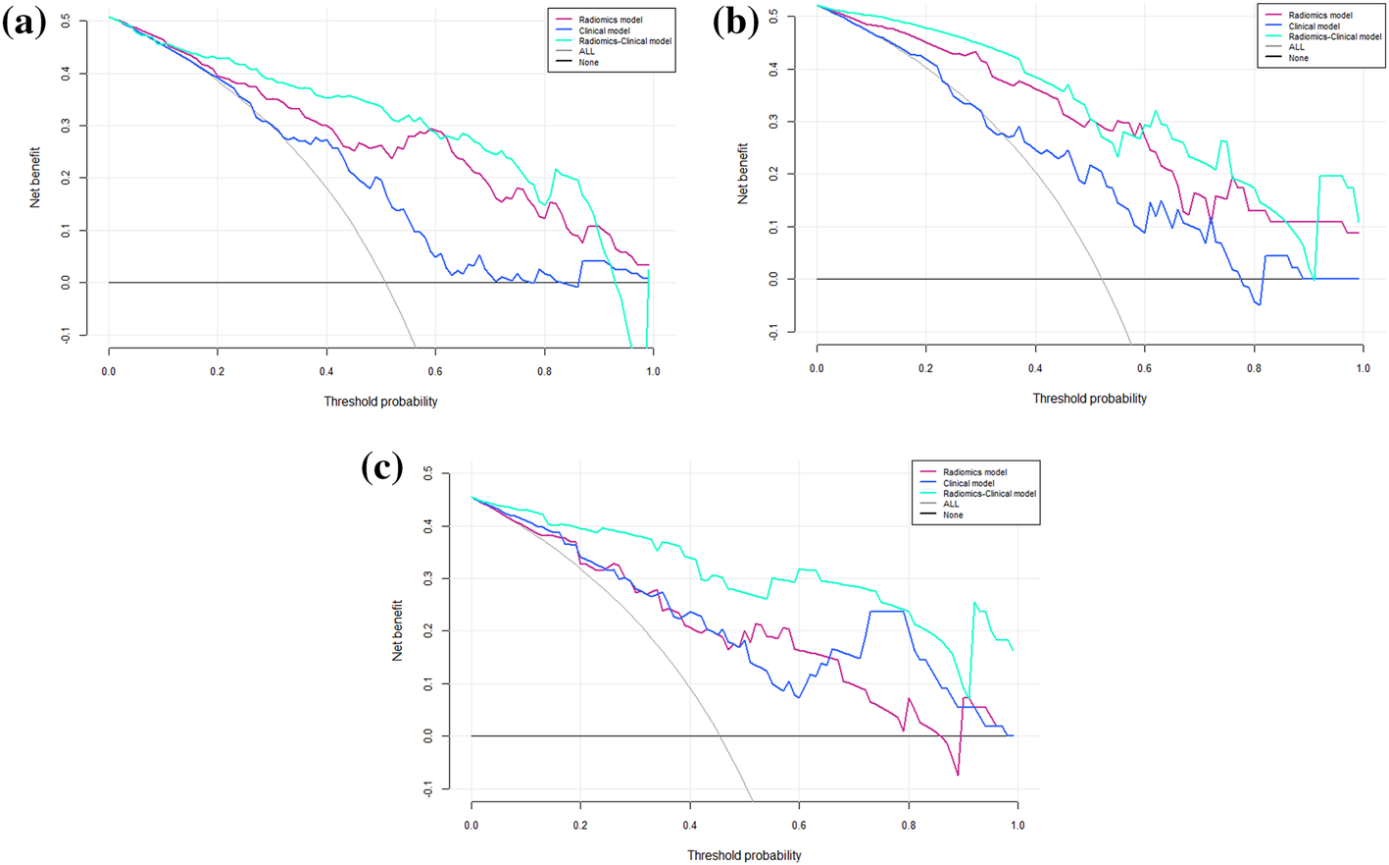
DCA curve of clinical application assessment for **a** the training cohort, **b** the internal test cohort, and **c** the external test cohort. The *x*-axis shows the threshold probability, and the *y*-axis measures the net benefit. The gray line and the black line indicate that all patients receive the “all treatment” or “no treatment” strategy, respectively. The red line indicating the radiomics model; the blue line the clinical model; the green line the radiomics–clinical

Decision curve analysis is a method of describing clinical impact in terms of net benefits, the “treat all” or “treat none” strategy is interpreted here as the net benefit achieved by all patients receiving or not receiving HIFU treatment. DCA analysis results show that the combined model achieves more net benefit across the majority of the range of threshold probabilities compared with the radiomics model, clinical model, treat-all strategy, and treat-none strategy (Fig. 5).

## Discussion

Here we developed a radiomics–clinical prediction model through SVM algorithm based on 7 radiomics features and 5 clinical parameters to predict the NPVR of uterine fibroids treated by HIFU. This model was evaluated in the internal and external test cohorts, demonstrating excellent prediction performance (AUC = 0.824, 0.773, respectively). The decision curve analysis also showed this model had the highest overall net benefit among the 3 models established in this study, which means that the model will contribute to clinical decision making.

The signal intensity can reflect proliferative activity and histological characteristics, such as cellularity, vascularity, perfusion, necrosis, and calcification [21, 22]. Therefore, the Funaki classification base on T2 signal intensity is a well-known predictor of the immediate therapeutic responses to MR-guided HIFU ablation therapy. A recent study results utilizing ultrasound-guided HIFU demonstrated a similar influence of Funaki type on NPVR as reported for MRgHIFU: the NPVR of Funaki type 1 and 2 was significantly higher than that of Funaki type 3 [23]. Funaki type 3 fibroids tend to display high cellularity related to their fiber content, edema or degeneration, making heat accumulation difficult [24]. In this study, a clinical model was established by combining the T2 signal intensity with some meaningful clinical features that predict NPVR (including fat thickness, fibroids volume, fibroid diameter and distance from fibroids to the skin surface [25, 26]). The AUC values of this model were 0.714 and 0.699, respectively, in the internal and external test cohorts. The reason for the higher predictive performance than previous studies [10] may be related to our inclusion criteria, because only the fibroids located in the anterior wall with grade 1 or 2 blood supply were enrolled. The patients with anterior fibroids have shorter acoustic pathways, so the ultrasound attenuation is less as penetrating fewer tissue layers [27]. Allowing for the difficulties with obtaining the quantitative parameters of the blood perfusion, the influence of blood perfusion was not considered in this study, and only the fibroids with the blood supply of grade 1 or 2 were selected, which have potentially better therapeutic outcomes [28]. This strategy helps us exclude fibroids that are rich in the blood supply. Despite its better performance than previous reports, the limitations of the clinical model were still observed in our study, possibly due to the fact that the inherent heterogeneity of fibroids was ignored.

Some studies have pointed out that the subjective nature of T2WI interpretation and difficulty of differentiating the actual tissue or condition causing the relative signal intensity variations are limitations of the Funaki classification, and due to the relatively weak correlations and large overlap in NPV ratios between groups, T2-based images may not be the optimal predictor of treatment outcome [29, 30]. Radiomics provides accurate quantitative information for characterizing the heterogeneity of fibroids by quantifying the small structural differences in images caused by different histopathologies of tumors [31–33]. Su [34] thought that the texture features of T2MRI could demonstrate the cellular structure of uterine fibroids and could be used to predict immediate NPVR. Arnaud [35] multivariate analysis results also revealed that quantitative analysis of texture showed a higher significant correlation with treatment success than qualitative classification, which may interpret why the predictive validity of our radiomics model is superior to the clinical model based on Funaki classification. The radiomics–clinical model established on combining clinical parameters with radiomics features demonstrates the strongest predictive ability in both the internal and the external test cohorts. This result indicates that the analysis of the combination of radiomics and clinical parameters is valuable and necessary for the prediction of NPVR, and the DCA also showed that the combined model would allow more patients to benefit from accurately predicting NPVR compared with the radiomics or the clinical model alone across the majority of the range of reasonable threshold probabilities. This is consistent with the results of a previous study on the prognosis of uterine fibroids [20].

An important goal of radiomics is to construct prediction models of therapeutic response based on tumor phenotypic characteristics from medical images, our research results indicate that it is promising to predict the NPVR of HIFU in the treatment of uterine fibroids using radiomics features, this is an objective quantitative measurement method that is closely related to NPVR, and shows better predictive ability than Funaki classification. In addition, we included patients from different institutions and MRI facilities, multicenter external validation is essential to obtain high-level evidence for future clinical applications, but a major problem faced by multicenter validation is that MRI intensities are non-standardized and are highly dependent on the manufacturer, sequence type and acquisition parameters. Consequently, a large variability in image intensities among inter-patient and intra-patient acquisitions, which may compromise the robustness of the radiomics features [36, 37]. To solve this problem, we performed image preprocessing such as resampling and intensity normalization to mitigate the effects that may occur due to heterogeneity of these MRI acquisitions. We believe that our results showed the potential of radiomics for NPVR prediction even using MRI with heterogeneous protocols. Therefore, our model is more beneficial to generalize in future clinical application and support the clinical transfer in different hospitals.

Our study had some limitations. First, this was a retrospective study, so inherent biases and variations were inevitable, and a prospective study may be conducted in the future for further verification. Second, only radiomics features from T2MRI were used in this study, while the application of multimodal studies such as T1-weight images and DWI sequences may improve the predictive performance. Third, we did not try to use other NPVR values as prediction dividing lines for treatment success, only NPVR ≥ 80% was used in this study. Because measures to achieve 80% NPVR is technically feasible and have been proven to be highly associated with a clinical treatment success [3, 4, 38]. Finally, while patients at both institutions were treated with the same HIFU system, a potential biased limitation is that the treatment was performed by different doctors, which could influenced dosage delivery or the termination of treatment. In addition, our screening parameters only include clinical and radiomics features. Future studies must also include HIFU treatment parameters, including irradiation mode, irradiation time, sound power, cooling time between each sonication, etc.

## Conclusion

The research results show that the combination model has the best prediction performance and the potential to conduct multicentre studies. In future studies, the model would benefit from more detailed and accurate division and modelling of uterine fibroids to reduce the factors influencing NPVR prediction and improve predictive accuracy.

## Materials and methods

### Patients

This retrospective study was approved by the Ethics Committees of the First Affiliated Hospital of Chongqing Medical University and Chongqing Haifu Hospital and conducted in accordance with the Declaration of Helsinki. Patients diagnosed with uterine fibroids by MRI and clinical examination from February 2012 to July 2020 were enrolled in this two-center retrospective study. The inclusion criteria for this study were as follows: (1) age of 18 years or older with clinical symptoms; (2) fibroids with the blood supply of grade 1 or grade 2 by the Adler classification using color doppler ultrasound examinations [39]; (3) no history of HIFU treatment or surgical resection. (4) safe and clear acoustic pathway with no scar tissue in the abdomen; (5) no more than two fibroids in each patient, determined by MRI. The exclusion criteria were as follows: (1) patients have contraindications for MRI examination and contrast injection; (2) fibroids are not located on the anterior wall of the uterus. (3) Missing preoperative and postoperative MR images, or image has artifacts.

The patients had from 1 to 2 uterine fibroid tumors; however, only the largest fibroids treated were selected for this study. A total of 223 patients included in this retrospective analysis. 168 patients from the First Affiliated Hospital of Chongqing Medical University were randomly assigned to the training cohort (*n* = 122) and the internal test cohort (*n* = 46), and 55 patients from Chongqing Haifu Hospital were selected as external testing cohort. All the patients were divided into 2 groups according to their immediate postoperative NPVR value in this study, among whom patients with NPVR ≥ 80% were assigned to group H, while those with NPVR < 80% were entered into group L.

### HIFU ablation

HIFU ablation was performed by HIFU-licensed physicians with at least 3 years of HIFU clinical experience, and the ultrasound-guided HIFU system (Model-JC200 Focused Ultrasound Tumor Therapeutic System, Chongqing Haifu Medical Technology Co., Ltd, Chongqing, China) was employed in both centers. Colour Doppler ultrasonography was performed with a My-Lab 70 ultrasound imaging device (Esaote, Genoa, Italy), which is located at the center of the high-intensity focused ultrasound transducer. The frequency of the transabdominal ultrasound probe was 3.5 MHz, the depth range 15 cm, the sampling volume 2–3 mm, and the angle between the sound beam and the blood flow less than 60 degrees. Before the treatment the patients were positioned prone on the HIFU table with the anterior abdominal wall placed in contact with the degassed water, a urinary catheter was inserted into the bladder, and normal saline was used to regulate the bladder volume to get a safe acoustic pathway. Treatment began from the deeper part of the fibroid to the shallower part with the focus at least 1 cm away from the boundary of the fibroid, and the therapeutic energy was adjusted based on the feedback from the patient and changes in grayscale on ultrasonographic imaging. The treatment was terminated when the increased grayscale covered the fibroid or there was an absence of blood supply as assessed by contrast-enhanced ultrasound immediately after HIFU ablation.

### Data collection

All patient’s data were evaluated by at least two experienced abdominal radiologists, such as subcutaneous fat thickness (mm), distance from fibroids to the skin surface (mm), fibroids volume (mm^3^) and T2 signal intensity. The T2 signal intensity of uterine fibroids was classified according to relevant studies into four types [7, 8]: (i) hypointense; (ii) isointense; (iii) heterogeneous hyper-intense; and (iv) homogenous hyper-intense. MRI was performed to assess the therapeutic outcomes within 1 days after HIFU treatment, the three-dimensional diameter of the volume of fibroids and non-perfused volume (NPV) were measured by enhanced T1-weighted image (T1WI) after treatment: longitudinal (D1), anteroposterior (D2), and transverse (D3). Fibroid volume and NPV were calculated according to the following equation: *V* = 0.5233 × *D*1 × *D*2 × *D*3, and the NPVR was defined as NPV/*V*_fibroids_ × 100%.

### MRI acquisition and segmentation

A series of standard T1 weighted images (T1WI) and T2 weighted images (T2WI) and contrast-enhanced T1WI scan were performed on all patients 1 day before and 1 day after the treatment. Dynamic enhancement was performed 20 s after an intravenous injection of gadolinium. Sagittal T2WI was selected as the object of radiomics analysis. In the First Affiliated Hospital of Chongqing Medical University, MRI was performed using 3.0-T scanners (Signa HDxT, GE healthcare), and MRI was performed using a 1.5-T scanner (uMR 570, United Imaging Company) at Chongqing Haifu Hospital. The T2 imaging parameters of the two participating centers are shown in Table 3.

**Table 3:** T2-weighted imaging parameters used by different scanners at the two participating centers

Variable	The First Affiliated Hospital of Chongqing Medical University	Chongqing Haifu Hospital
Repetition time (ms)	3400–5100	3200–4200
Echo time (ms)	110	79
Field of view (cm × cm)	36 × 36	22 × 22
Matrix size (mm × mm)	512 × 512	320 × 320
Slice thickness (mm)	6	5
Slice gap (mm)	1.5	1.0

**Fig. 6:**
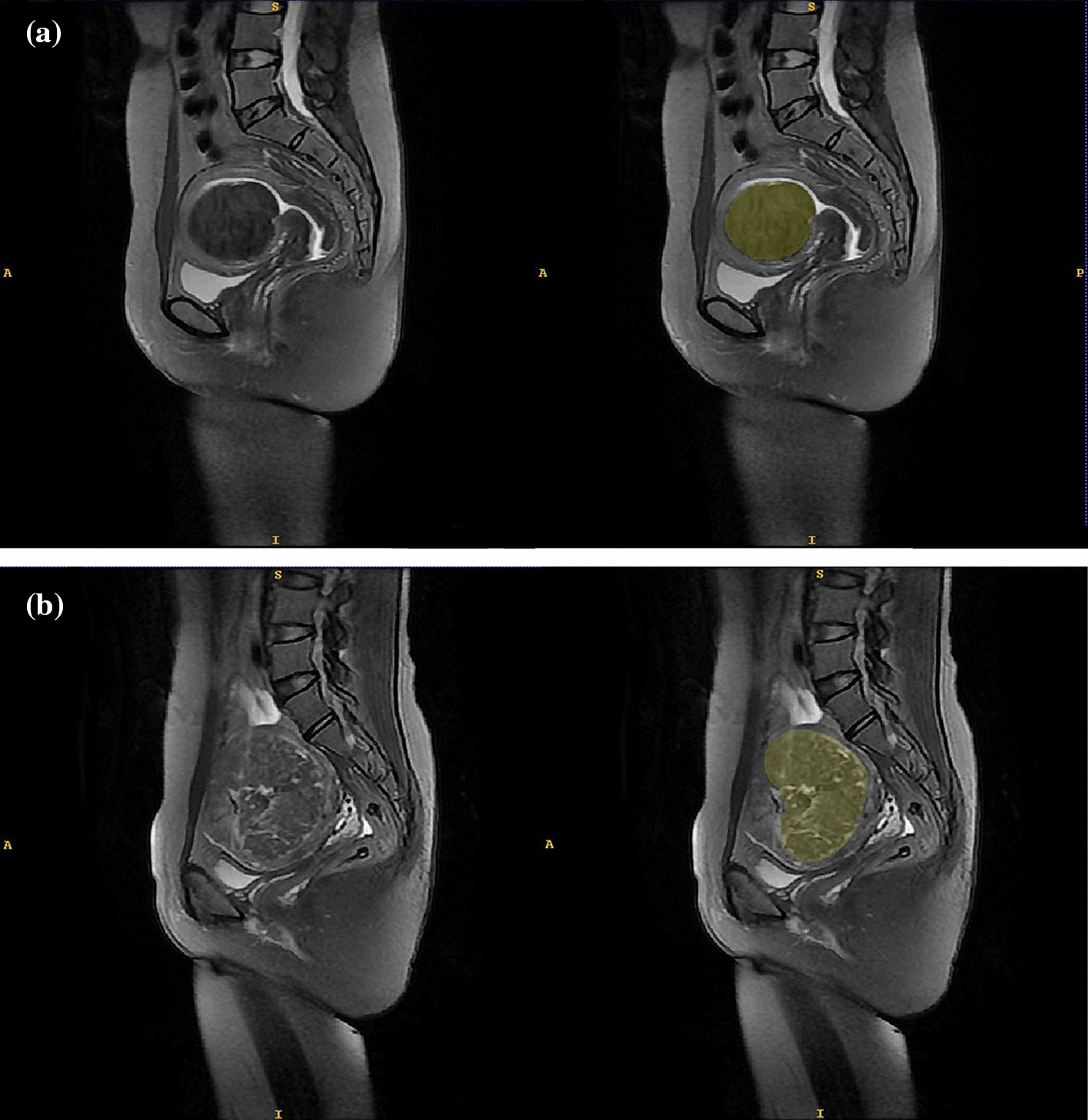
Example of T2-weighted magnetic resonance images (left) and tumor segmentation (right) of uterine fibroids obtained before treatment. **a** Hypo-intense fibroid; **b** heterogeneous hyper-intense fibroid

An abdominal radiologist (reader 1) with 5 years of experience in pelvic radiological imaging was responsible for delimiting the region of interest (ROI) slice by slice along the fibroid edge on the T2MRI for each patient using open source software (ITK-SNAP v3.8.0, www.itksnap.org), as shown in Fig. 6. Each labeled segmentation was validated by another senior radiologist (reader 2) with a 14-year career in pelvic radiological imaging.

### Image preprocessing and feature extraction

Image processing was performed prior to feature extraction to minimize the variance induced by different scanners, scanning scheme as well as acquisition artifacts. First, the N4ITK bias field correction algorithm was applied to all images to reduce image artifacts and improve the inhomogeneity of grayscale distribution [40]. Next, all images were normalized using *z*-score normalization to obtain a standard normal distribution of image intensity. Finally, B-spline interpolation was adopted to set the voxel size at 1 × 1 × 1 mm^3^ for resampling. More details of image processing are shown in Additional file 1: Table S1.

Radiomics features of each ROI were extracted from T2WI with Pyradiomics v.3.0 package, and feature extraction followed the Image Biomarker Standardization Initiative (IBSI) guideline in this study [41]. The extracted features include shape (3D), first-order statistics, gray-level co-occurrence matrix (GLCM), gray-level size zone matrix (GLSZM), gray-level run length matrix (GLRLM), neighboring gray-tone difference matrix (NGTDM), gray-level dependence matrix (GLDM), and wavelet features. The details are shown in Additional file 1: Table S2.

### Reproducibility analysis and feature selection

To assess the reproducibility of radiomics features, thirty images were randomly selected for ROI segmentation by two radiologists (reader1 and reader 2). Intraclass correlation coefficients (ICCs) were calculated to assess the reproducibility of the radiomics features extracted from all the ROI drawn by the two radiologists, and features with an ICC ≥ 0.8 were considered reliable.

All radiomics features were standardized by the z-score method. The least absolute shrinkage and selection operator (LASSO) regression was applied to identify and select the optimal radiomics features in the training cohort. Tenfold cross-test method was used to determine the best value of parameter *λ*. According to the non-zero coefficient features selected by LASSO, the individual radiomics score (Rad-score) was calculated by a linear combination of each feature weighted by its respective coefficients.

### Model construction and evaluation

The clinical model, the radiomics model, and the radiomics–clinical model were established according to clinical parameters, radiomics features, and radiomics features together with clinical parameters, respectively. Support Vector Machines (SVM) was adopted to construct the model (Python scikit-learn environment, version 0.21.3). In the training cohort, these classification models were trained using tenfold cross-validation, and grid search was used for hyperparameter tuning, including “gamma”, “C” and kernel function. Additional file 1: Table S3 documents the hyperparameters of the different models.

The model was validated in both the internal and external test cohort, and the predictive performance of the model was assessed in different cohorts using the area under the curve (AUC) analysis, A nonparametric bootstrap method was used to calculate the 95% confidence interval (CI) by the repeated 2000 times sampling in three cohorts. Decision curve analysis (DCA) was also performed to evaluate whether the prediction model contributes to clinical treatment strategies by calculating the net benefits of the model at different threshold probabilities in the 3 cohorts (training, internal, and external test).

### Statistical analysis

All statistical analyses were performed with SPSS 19.0 (IBM, Armonk, NY), and the data distribution was tested using Kolmogorov–Smirnov analysis. Frequencies and percentages were expressed as categorical variables, while the mean and standard deviation as continuous variables. Continuous variables were compared by the independent samples *t *test or the Mann–Whitney *U* test, and the categorical variables by Chi*-*square test. The level of statistically significant difference was set at *P* < 0.05.

### Supplementary Information

**Additional file 1: Table S1.** Feature extraction configuration following the guidelines of the Image Biomarker Standardization Initiative (IBSI) in this study. **Table S2.** Extracted features were classified into eight categories as follows. **Formula S1.** The formula of Rad-scores derived from LASSO. **Table S3.** Hyperparameters corresponding to different models trained by SVM.** Table S4.** Comparison of Rad-scores for patients with NPVR ≥ 80% and patients with < 80%.

## Data Availability

The data sets and source code used and/or analyzed during the current study are available from the corresponding author on reasonable request.

## Abbreviations

T2MRI: T2-weighted magnetic resonance imaging
NPVR: Non-perfused volume ratio
USgHIFU: Ultrasound-guided high-intensity focused ultrasound
LASSO: Least absolute shrinkage and selection operator
SVM: Support vector machine algorithm
ROI: Region of interest
AUC: Area under the ROC curve
GLCM: Gray-level co-occurrence matrix
GLDM: Gray-level dependence matrix
GLRLM: Gray-level run length matrix
GLSZM: Gray-level size zone matrix
NGTDM: Neighboring gray tone difference matrix
3D: Three-dimensional
DCE-MRI: Dynamic contrast-enhanced MRI
