# Supplementary material for: Prediction of non-perfusion volume ratio for uterine fibroids treated with ultrasound-guided high-intensity focused ultrasound based on MRI radiomics combined with clinical parameters
Source: Biomed Eng Online. 2023 Dec 13;22:123. doi: 10.1186/s12938-023-01182-z (PMC10717163; doi:10.1186/s12938-023-01182-z)
Supplement: Supplementary file 1 — Additional file 1: Table S1. Feature extraction configuration following the guidelines of the Image Biomarker Standardization Initiative (IBSI) in this study. Table S2. Extracted features were classified into eight categories as follows. Formula S1. The formula of Rad-scores derived from LASSO. Table S3. Hyperparameters corresponding to different models trained by SVM. Table S4. Comparison of Rad-scores for patients with NPVR ≥ 80% and patients with < 80%. [file 12938_2023_1182_MOESM1_ESM.docx]

**Table S1.** The feature extraction configuration following the guidelines of the Image Biomarker Standardization Initiative (IBSI) in this study

| Parameter |  | Config | | | |
| --- | --- | --- | --- | --- | --- |
| Slice-wise or singlevolume (3D) |  | 3D | | | |
| Interpolation |  |  | | | |
|  | Resampled voxel spacing (mm) | 1 × 1 × 1 | | | |
|  | ROI interpolation method | B Spline | | | |
| Re-segmentation |  |  | | | |
|  | Range | [μ －3σ, μ ＋3σ] | | | |
|  | Outlier filtering | no | | | |
| Discretisation |  |  | | | |
|  | Texture and intensity-based statistics features | FBS: 25 | | | |
| Features parameters |  | **Distance** | **Count** | **ROI mask** | |
|  |  |  |  | Morph. | Int. |
|  | Morphology | **×** | **14** | **√** | **×** |
|  | Intensity-based statistics | **×** | **18** | **×** | **√** |
|  | GLCM | **1** | **24** | **×** | **√** |
|  | GLRLM | **1** | **16** | **×** | **√** |
|  | GLSZM | **1** | **16** | **×** | **√** |
|  | GLDM | **1** | **14** | **×** | **√** |
|  | NGTDM | **1** | **5** | **×** | **√** |

μ: the mean of the ROI; σ: the standard deviations of the ROI.

Morph：morphological mask; Int: intensity mask;

**Table S2.** The extracted features were classified into eight categories as follows:

| 1.First Order Statistics (18 features) | Energy; Total Energy; Entropy; Minimum; 10th percentile; 90th percentile; Maximum; Mean; Median; Interquartile Range; Range; Mean Absolute Deviation (MAD); Robust Mean Absolute Deviation (rMAD); Root Mean Squared (RMS); Skewness; Kurtosis; Variance; Uniformity; |
| --- | --- |
| 2.Shape–based (3D) (14 features) | Mesh Volume; Voxel Volume; Surface Area; Surface Area to Volume ratio; Sphericity; Maximum 3D diameter; Maximum 2D diameter (Slice); Maximum 2D diameter (Column); Maximum 2D diameter (Row); Major Axis Length; Minor Axis Length; Least Axis Length; Elongation; Flatness; |
| 3.Gray Level Cooccurence Matrix (24 features) | Autocorrelation; Joint Average; Cluster Prominence; Cluster Shade; Cluster Tendency; Contrast; Correlation; Difference Average; Difference Entropy; Difference Variance; Joint Energy; Joint Entropy; Informational Measure of Correlation (IMC) 1; Informational Measure of Correlation (IMC) 2; Inverse Difference Moment (IDM); Maximal Correlation Coefficient (MCC); Inverse Difference Moment Normalized (IDMN); Inverse Difference (ID); Inverse Difference Normalized (IDN); Inverse Variance; Maximum Probability; Sum Average; Sum Entropy; Sum of Squares; |
| 4.Gray Level Size Zone Matrix (16 features) | Short Run Emphasis (SRE); Long Run Emphasis (LRE); Gray Level Non-Uniformity (GLN); Gray Level Non-Uniformity Normalized (GLNN); Run Length Non-Uniformity (RLN); Run Length Non-Uniformity Normalized (RLNN); Run Percentage (RP); Gray Level Variance (GLV); Run Variance (RV); Run Entropy (RE); Low Gray Level Run Emphasis (LGLRE); High Gray Level Run Emphasis (HGLRE); Short Run Low Gray Level Emphasis (SRLGLE); Short Run High Gray Level Emphasis (SRHGLE); Long Run Low Gray Level Emphasis (LRLGLE); Long Run High Gray Level Emphasis (LRHGLE); |
| 5.Gray Level Run Length Matrix (16 features) | Small Area Emphasis (SAE); Large Area Emphasis (LAE); Gray Level Non-Uniformity (GLN); Gray Level Non-Uniformity Normalized (GLNN); Size-Zone Non-Uniformity (SZN); Size-Zone Non-Uniformity Normalized (SZNN); Zone Percentage (ZP); Gray Level Variance (GLV); Zone Variance (ZV); Zone Entropy (ZE); Low Gray Level Zone Emphasis (LGLZE); High Gray Level Zone Emphasis (HGLZE); Small Area Low Gray Level Emphasis (SALGLE); Small Area High Gray Level Emphasis (SAHGLE); Large Area Low Gray Level Emphasis (LALGLE); Large Area High Gray Level Emphasis (LAHGLE); |
| 6.Gray Level Dependence Matrix (14 features) | Small Dependence Emphasis (SDE); Large Dependence Emphasis (LDE); Gray Level Non-Uniformity (GLN); Dependence Non-Uniformity (DN); Dependence Non-Uniformity Normalized (DNN); Gray Level Variance (GLV); Dependence Variance (DV); Dependence Entropy (DE); Low Gray Level Emphasis (LGLE); High Gray Level Emphasis (HGLE); Small Dependence Low Gray Level Emphasis (SDLGLE); Small Dependence High Gray Level Emphasis (SDHGLE); Large Dependence Low Gray Level Emphasis (LDLGLE); Large Dependence High Gray Level Emphasis (LDHGLE); |
| 7.Neighbouring Gray Tone Difference Matrix (5 features) | Coarseness; Contrast; Busyness; Complexity; Strength; |
| 8. Wavelet Features (744 features) | wavelet-LLH, wavelet-LHL, wavelet-LHH, wavelet-HLH, wavelet-HLL, wavelet-LLL, wavelet-HHL, wavelet-HHH |

**formula S1.** The formula of Rad-scores derived from LASSO:

**Table S3.** Hyperparameters corresponding to different models trained by SVM.

| ***Model***  ***Hyperparameters*** | **Radiomics** | **Clinical** | **Combination** |
| --- | --- | --- | --- |
| **hyperparameters-tuning range** | **kernel function**: [linear, rbf, sigmoid, poly]  **Gamma**: [0.05, 0.5, 1, 2, 5, 10]  **C**: [0.001, 0.002, 0.02, 0.025, 0.2, 0.25, 0.5, 1, 5, 10] | | |
| **kernel function** | “linear” | “linear” | “linear” |
| **Gamma** | 0.05 | 0.05 | 0.05 |
| **C** | 0.025 | 0.5 | 0.02 |

**Table S4.** Comparison of Rad-scores for patients with NPVR ≥ 80% and patients with < 80%

|  | Group H: NPVR ≥ 80% | Group L: NPVR < 80% | *P* value |
| --- | --- | --- | --- |
| Training cohort | 0.420±0.121 | 0.593±0.147 | **0.000*** |
| Internal test cohort | 0.416±0.127 | 0.593±0.134 | **0.000*** |
| External test cohort | 0.445±0.123 | 0.589±0.170 | **0.001*** |
| Independent samples *t* test or the Mann–Whitney *U* test were used to compare the differences in Rad-scores between the two groups, *P<0.05 means statistical significance. | | | |
